# Heterogeneous Versus Homogeneous Radiation Dose Calculations of Twice-Daily Fractionation in Small Cell Lung Carcinoma

**DOI:** 10.7759/cureus.20226

**Published:** 2021-12-07

**Authors:** Ryan Thibodeau, Hsin K Li, Sean Tanny, Ajeet Gajra, Jeffrey Bogart

**Affiliations:** 1 Department of Radiation Oncology, State University of New York Upstate Medical University, Syracuse, USA; 2 Department of Radiation Oncology, State University of New York Upstate Medical University, Syracuse, USA; 3 Department of Radiation Oncology, University of Rochester Medical Center, Rochester, USA; 4 Department of Medical Oncology, State University of New York Upstate Medical University, Syracuse, USA

**Keywords:** radiation oncology, medical physics, dosimetry, small cell lung cancer, dose calculation, three-dimensional conformal radiotherapy, intensity-modulated radiation therapy

## Abstract

Purpose

The standard radiotherapy regimen for small cell lung cancer (SCLC) was determined using dose calculations without corrections for tissue heterogeneity, while modern treatments are planned using algorithms accounting for tissue heterogeneity. We assessed differences in dose delivered using heterogeneous and homogeneous dose calculations in a cohort of patients treated for limited-stage small cell lung cancer (LS-SCLC).

Methods

This is a retrospective analysis of 35 patients (three-dimensional conformal radiation therapy (3D-CRT), n = 22; intensity-modulated radiation therapy (IMRT), n = 13) with LS-SCLC treated with chemoradiotherapy from 2011 to 2017. Treatment plans were developed in the Eclipse Treatment Planning System (TPS) version 13.6 using the Analytical Anisotropic Algorithm (AAA). Two plans were generated for each patient with one using the unit relative electron density and the other maintaining the same monitor units (MUs) with tissue density corrections. The prescription was 45 Gy in 30 fractions of 1.5 Gy delivered twice daily. Individuals who underwent replanning within the same treatment course were evaluated using a separate corrected and uncorrected plan sum. Variations greater than 5% in dose to the tumor or organs at risk were considered clinically relevant. A two-sided paired t-test was used to evaluate the statistical significance of the dosimetric differences.

Results

The percent dose difference between plans without tissue heterogeneity corrections to those with corrections resulted in an overall median difference of -3% (range: -15.1% to 9.6%; p < 0.01) for the dose covering 95% of the planning target volume (PTV D95) and was -5.6% (range: -17.3% to 5.4%; p < 0.01) for lung volume receiving ≥20 Gy (lung V20). For 3D-CRT, the median difference for the PTV D95 was -0.1% (range: -4.7% to 9.6%; p = 0.62) and the lung V20 was -4.2% (range: -9.4 to 5.4; p < 0.01). For IMRT, the median difference for the PTV D95 was -10.0% (range: -15.1% to -5.3%; p < 0.01) and the lung V20 was -8.9% (range: -17.3 to -3.5; p < 0.01).

Conclusion

Traditional planning without tissue heterogeneity corrections results in an overall decrease in the dose delivered to the target compared with those that incorporate tissue heterogeneity corrections. These differences are modest for 3D treatment plans but may result in clinically relevant differences for the IMRT cohort (>5% deviation).

## Introduction

Lung cancer is the leading cause of cancer-related deaths in the United States for both men and women in 2019. Approximately 230,000 new cases of lung cancer are diagnosed each year with an associated mortality rate of nearly 25% [1]. Small cell lung cancer (SCLC) accounts for nearly 15% of all lung cancer cases. The majority of individuals have metastatic disease at presentation and are more commonly referred to as having extensive-stage small cell lung cancer. Only 30% of patients will be diagnosed with limited-stage small cell lung cancer (LS-SCLC) [2-4]. This staging system was defined by the Veterans Administration Lung Group in accordance with whether the disease is confined to one hemithorax and could be adequately encompassed in the radiation field [4].

Chemoradiotherapy is the mainstay of treatment for limited-stage small cell lung cancer (LS-SCLC) with demonstrated improvements in overall survival in comparison with chemotherapy alone [5-7]. Radiotherapy is commonly initiated with either the first or second cycle of chemotherapy. Turrisi et al. established the current standard of care with significant improvement in two- and five-year survival rates with cisplatin plus etoposide with 45 Gy delivered twice daily at least six hours apart in 30 fractions when compared with 4500 cGy delivered once daily over 25 fractions [8]. Following this, Bogart et al. showed that delivering 70 Gy in 35 once-daily fractions with concurrent chemotherapy could be performed safely with preliminary data suggesting comparable results to 45 Gy delivered twice daily in 30 fractions [9]. This particular treatment regimen was the subject of a phase III clinical trial (CALGB 30610), which is now reported to show a favorable comparison between the two regimens [10]. Recently, Faivre-Finn et al. published results from the CONVERT trial that demonstrated that individuals with LS-SCLC treated with 45 Gy in twice-daily fractions in comparison with 66 Gy in once-daily fractions with concurrent chemotherapy compared favorably with historical outcomes and did not significantly differ in patients’ overall survival [11].

SCLC often occurs in close proximity to and/or involves the mediastinum [12]. Traditional 3D treatment plans usually combine an anterior-posterior/posterior-anterior (AP/PA) field arrangement to 30 Gy, followed by an off-cord boost for the remaining 15 Gy. Compared with traditional 2D treatment planning, 3D treatment plans grant the ability to incorporate tissue heterogeneity calculations, although they do not typically evaluate target motion using fluoroscopy or four-dimensional computed tomography (4D-CT) methods. Modern techniques, such as intensity-modulated radiation therapy (IMRT) and volumetric modulated arc therapy (VMAT) techniques, are generally planned on an average projection image from a 4D-CT.

To date, there are no published studies evaluating the impact of heterogeneity correction or IMRT/VMAT on radiotherapy plans relative to those delivered to patients in the seminal trial by Turrisi et al. [8]. This study was performed without heterogeneity corrections per INT0096/RTOG 88-15 protocol, which was typical of the time [13]. Therefore, we evaluated the differences between homogeneous and heterogeneous dose calculations in 35 patients and the impact of 3D versus IMRT/VMAT treatment techniques on those differences. We wanted to determine the differences in dose delivered by modern treatment techniques with heterogeneity corrections compared with doses that were delivered establishing the standard of care for LS-SCLC.

The abstract of this article has been presented at the Latin American Conference on Lung Cancer in November 2019 [14].

## Materials and methods

Study cohort

We retrospectively reviewed 35 individuals who underwent definitive chemoradiotherapy at our institution for LS-SCLC using twice-daily radiation treatments between January 1, 2010, and December 31, 2017, queried from the Eclipse Treatment Planning System (TPS) version 13.6 (Varian Medical Systems, Palo Alto, CA, USA). Institutional review board approval was obtained for this study. Demographic and dosimetric information was collected. The patients’ radiation treatment plans were recalculated using the Analytical Anisotropic Algorithm (AAA). Each treatment plan was calculated with and without tissue heterogeneity corrections. As all individuals in this treatment cohort were originally treated with plans that accounted for heterogeneity, two plans were generated for each patient with one using the unit density and another plan with the same monitor units (MUs) and applying tissue heterogeneity corrections. Dosimetric data were collected for all plans and compared. The prescription dose was 45 Gy in 30 fractions of 1.5 Gy twice daily separated by at least six hours. Individuals who underwent replanning within the same treatment course were evaluated using a separate corrected and uncorrected plan sum. Treatment plans were initially normalized such that 95% of the planning target volume (PTV) was covered by 100% of the prescription dose.

Dosimetric measures and statistical analysis

Dosimetric information collected for each treatment plan included esophagus mean, heart max, spinal cord max, lung mean, lung volume receiving ≥20 Gy (lung V20), dose covering 99% of the gross tumor volume (GTV D99), GTV min, GTV max, dose covering 95% of the planning target volume (PTV D95), PTV min, and PTV max. Treatment plans were stratified by whether they were treated using three-dimensional conformal radiation therapy (3D-CRT) versus IMRT/VMAT treatments and by treatment energy. Dosimetric differences were calculated as seen in Equation 1, where $D_{Homo}$ is the dose without heterogeneity corrections and $D_{Hetero}$ is the dose with heterogeneity corrections:

$\% Difference = \frac{D_{Homo} - D_{Hetero}}{D_{Hetero}} \times 100$ (1)

According to International Commission on Radiation Units and Measurements Report 24 and a recent International Atomic Energy Association report on uncertainty in radiation therapy dose, tumor dose differences > 5% from intended prescription dose would be classified as clinically significant differences [15,16]. Two-tailed, paired t-tests using the IBM Statistical Package for the Social Sciences version 26 (Armonk, NY, USA) were computed to compare the dosimetric differences between doses with and without heterogeneity correction.

## Results

Patient and treatment characteristics

We queried the TPS for individuals with LS-SCLC treated from January 1, 2010, to January 1, 2018, and a total of 35 patients were identified. Twenty-two patients received 3D-CRT treatments, and the remaining 13 patients received IMRT/VMAT treatments. Their demographic information are displayed in Table 1.

**Table 1:** Demographic Information ^a^Staging per the American Joint Committee on Cancer (AJCC) seventh edition staging manual ECOG = Eastern Cooperative Oncology Group

		3D-CRT (n = 22)	IMRT (n = 13)
Sex	Male	10/22 (54.5%)	6/13 (46.2%)
	Female	12/22 (45.5%)	7/13 (53.8%)
Age at Initial Consult (Years)	Mean (years)	61.8	64.8
	Median (years)	61.7	66.4
Stage^a^	Stage I	0	0
	Stage II	1/22 (4.5%)	1/13 (7.7%)
	Stage III	21/22 (95.5%)	12/13 (92.3%)
	Stage IV	0	0
ECOG Performance Status	0	4	4
	1	12	6
	2	5	2
	3	1	1

Dosimetric outcomes

**Figure 1:**
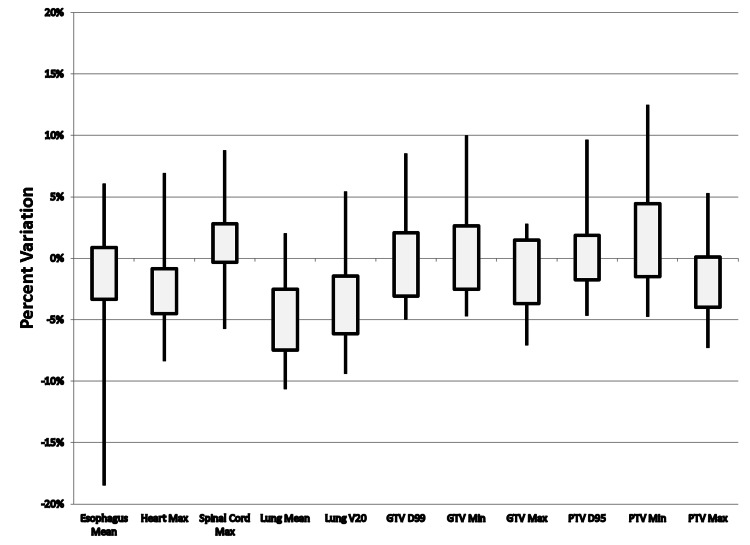
Variation in Dose Differences Between Heterogeneity Corrected Versus Heterogeneity Uncorrected Calculations for 3D-CRT Treatment Plans

**Figure 2:**
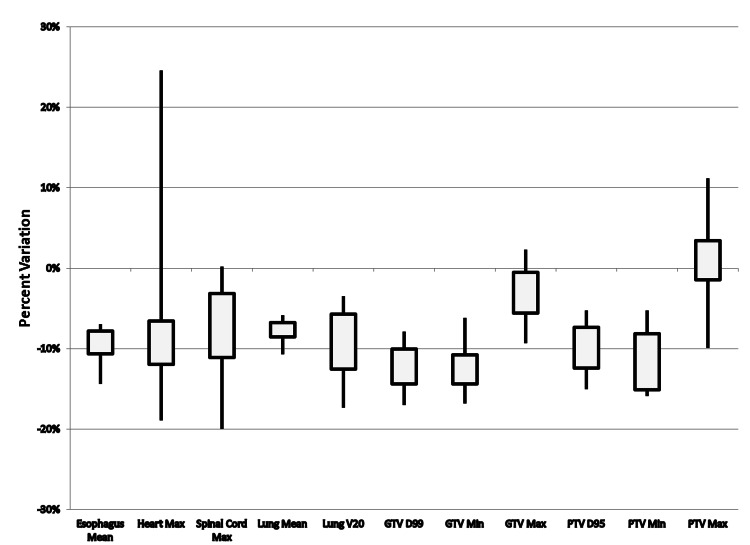
Variation in Dose Differences Between Heterogeneity Corrected Versus Heterogeneity Uncorrected Calculations for IMRT Treatment Plans

Significant dosimetric differences between heterogeneity corrected and homogeneous calculations for our cohort (35 patients) were observed involving the esophagus mean, heart max, spinal cord max, lung mean, lung V20, GTV D99, GTV min, GTV max, PTV D95, and PTV min. Clinically relevant differences (differences > 5%) though were primarily involving only the lung mean and lung V20. For individuals treated with 3D-CRT (22/35 patients), significant differences were found for heart max, lung mean, lung V20, GTV max, and PTV max. Clinically relevant differences for 3D-CRT were only seen for the lung mean. For patients treated with IMRT plans (13/35 patients), significant differences were found for esophagus mean, heart max, spinal cord max, lung mean, lung V20, GTV D99, GTV min, GTV max, PTV D95, and PTV min. Clinically relevant differences were found for IMRT plans involving the esophagus mean, heart max, spinal cord max, lung mean, lung V20, GTV D99, GTV min, PTV D95, and PTV min. Average deviations between dose calculations with/without tissue heterogeneity corrections are presented for the entire cohort in Table 2 and for 3D-CRT and IMRT plans in Table 3, respectively. The distributions of dose differences in 3D-CRT and IMRT plans for all patients are shown in Figure 1 and Figure 2, respectively.

**Table 2:** Overall Dosimetric Differences Between Tissue Heterogeneity Uncorrected Versus Tissue Heterogeneity Corrected Plans

			Overall Average Differences (%)	P-value
Esophagus Mean		Minimum	-18.5	<0.01
		Maximum	6.1	
		Median	-3.8	
Heart Max		Minimum	-18.9	<0.01
		Maximum	24.6	
		Median	-4.3	
Spinal Cord Max		Minimum	-20.0	0.02
		Maximum	8.8	
		Median	-0.5	
Lung Mean		Minimum	-10.7	<0.01
		Maximum	2.0	
		Median	-6.7	
Lung V20		Minimum	-17.3	<0.01
		Maximum	5.4	
		Median	-5.6	
GTV D99		Minimum	-17.0	<0.01
		Maximum	8.6	
		Median	-4.0	
GTV Min		Minimum	-16.8	<0.01
		Maximum	10.0	
		Median	-4.2	
GTV Max		Minimum	-9.4	0.03
		Maximum	2.8	
		Median	-2.8	
PTV D95		Minimum	-15.1	<0.01
		Maximum	9.6	
		Median	-3.0	
PTV Min		Minimum	-15.9	<0.01
		Maximum	12.5	
		Median	-2.9	
PTV Max		Minimum	-9.9	0.58
		Maximum	11.2	
		Median	-0.5	

**Table 3:** Dosimetric Differences Between Tissue Heterogeneity Uncorrected Versus Tissue Heterogeneity Correction for 3D-CRT and IMRT Two-tailed, paired t-tests were performed on 3D versus IMRT cohorts for each of the dosimetric parameters above.

			3D Average Differences (%)	P-value	IMRT Average Differences (%)	P-value
Esophagus Mean		Minimum	-18.5	0.20	-14.4	<0.01
		Maximum	6.1		-7.0	
		Median	-1.7		-8.3	
Heart Max		Minimum	-8.4	<0.01	-18.9	<0.01
		Maximum	6.9		24.6	
		Median	-2.6		-9.8	
Spinal Cord Max		Minimum	-5.8	0.12	-20.0	<0.01
		Maximum	8.8		0.2	
		Median	0.6		-6.6	
Lung Mean		Minimum	-10.7	<0.01	-10.7	<0.01
		Maximum	2.0		-5.9	
		Median	-5.4		-7.3	
Lung V20		Minimum	-9.4	<0.01	-17.3	<0.01
		Maximum	5.4		-3.5	
		Median	-4.2		-8.9	
GTV D99		Minimum	-5.0	0.86	-17.0	<0.01
		Maximum	8.6		-7.9	
		Median	-1.4		-13.4	
GTV Min		Minimum	-4.7	0.35	-16.8	<0.01
		Maximum	10.0		-6.2	
		Median	0.4		-13.2	
GTV Max		Minimum	-7.1	0.05	-9.4	<0.01
		Maximum	2.8		2.3	
		Median	-2.3		-2.9	
PTV D95		Minimum	-4.7	0.62	-15.1	<0.01
		Maximum	9.6		-5.3	
		Median	-0.1		-10.0	
PTV Min		Minimum	-4.8	0.07	-15.9	<0.01
		Maximum	12.5		-5.3	
		Median	2.3		-13.0	
PTV Max		Minimum	-7.3	0.04	-9.9	<0.01
		Maximum	5.3		11.2	
		Median	-1.3		1.1	

## Discussion

The purpose of our study was to assess the differences in dose delivered when accounting for heterogeneity corrections in individuals treated for LS-SCLC. The dose fractionation schema for this disease was determined with clinical trials that did not account for tissue heterogeneity. However, the difference in tissue density and thus electron density becomes significant in the thorax, as the lung tissue has been known to be approximately a third that of soft tissue [17]. The Compton effect, which is the primary therapeutic radiation-matter interaction, is influenced largely by electron density. This difference in electron density thus impacts differential dose absorption, resulting in tissue dose variability. Therefore, efforts need to be made to determine if historically calculated doses can be used to inform modern treatment planning that routinely incorporates tissue heterogeneity corrections.

The results of our study demonstrate that there are no statistically significant dosimetric differences between homogeneous and heterogeneous treatment plans overall. However, we found clinically relevant dose differences for the IMRT cohort (>5% median deviation), whereas this was not the case for the majority of the metric for the 3D treatment plans (<5% median deviation). We attribute this difference to the fact that IMRT plans typically use more beam angles or are entirely delivered by modulated arcs. This increased aperture approach increases the number of different paths that traverse the lungs and thereby increases the sensitivity of the plan to variations in path density.

These findings are consistent with previous work from Mizuno et al. who evaluated dose calculation with/without tissue heterogeneity corrections for 25 patients treated for stage III SCLC [18]. Their study evaluated dose calculation using superposition/convolution and AAA algorithms. Patients were treated to 40 Gy utilizing AP-PA fields, followed by a 20 Gy oblique off-cord boost. They found that the mean isocenter dose varied by 4% for the AP-PA portion of the treatment and 6% for the oblique off-cord boost portion. They also found maximum differences to the target volume of 9% and 11% for the AP-PA and off-cord boost treatments, respectively. This corresponds well with our results, where we found a maximum decrease with heterogeneity corrections in PTV D95 values of 9.6%. Both our study and Mizuno noted that these large variations are case-dependent. On average, dose calculation for 3D-CRT plans demonstrates agreement within 5% with or without tissue heterogeneity corrections applied.

Overall, our study suggests that the tumor volume would not be underdosed and that there are no clinically relevant differences in dose between homogeneous and heterogeneous treatment plans overall, which would support the continued use of 45 Gy in 30 fractions treated in twice-daily fractions in the context of modern treatment planning where heterogeneity correction is applied.

## Conclusions

We evaluated a cohort of 35 patients who received treatment for LS-SCLC (22 with 3D-CRT plans and 13 with IMRT/VMAT plans) with and without heterogeneity corrections. We found that traditional planning without heterogeneity corrections results in an overall decrease in dose delivered to target that we considered not clinically relevant (<5% median dose deviation). When evaluating patients by treatment modality and heterogeneity corrections, the differences were modest for 3D treatments (<5% median dose deviation) but could be clinically relevant (>5% median dose deviation) for IMRT/VMAT treatments. This reinforces the importance of proper treatment planning when utilizing advanced treatment techniques.
